# The Role of Tumor Debulking Surgery in Improving Survival of Patients with Head and Neck Cancer: A Systematic Review

**DOI:** 10.3390/curroncol33070409

**Published:** 2026-07-09

**Authors:** Aris I. Giotakis, Evangelos Tagkalos, Matthias Santer, Daniel Dejaco, Benedikt Hofauer

**Affiliations:** 1 Department of Otorhinolaryngology, Medical University of Innsbruck, 6020 Innsbruck, Austria; arisgiotakis@hippocratio.gr (A.I.G.); daniel.dejaco@tirol-kliniken.at (D.D.); benedikt-gabriel.hofauer@i-med.ac.at (B.H.); 2 Department of General, Visceral and Transplant Surgery, University Medical Center of the Johannes Gutenberg University Mainz, 55131 Mainz, Germany; vtagalos@gmail.com

**Keywords:** cytoreduction surgical procedures, debulking, head and neck neoplasms, disease-free survival, survival analysis, surgical procedures, operative

## Abstract

**Background/Objectives**: Data on the value of tumor debulking surgery are scarce. We aimed to examine whether tumor debulking surgery followed by non-surgical treatment (cases) improves survival compared to non-surgical treatment alone (controls) in patients with head and neck cancer (HNC). **Methods**: We performed a systematic review of studies published in the databases PubMed, Scopus and Cochrane Central Register of Controlled Trials up to 10 December 2024. Studies evaluating tumor debulking surgery followed by non-surgical treatment and reporting survival (local recurrence, disease-free survival or overall survival) in subjects with HNC were included; case reports were excluded. We assessed the quality of observational studies with the Newcastle–Ottawa Scale. **Results**: Among 11 retrospective small studies, three case–control studies (one of high quality, i.e., 7/9, and two of moderate quality, i.e., 6/9 and 5/9) suggested longer survival in 72 cases with predominantly squamous cell carcinoma (SCC) than in 40 controls with predominantly SCC. However, these survival benefits cannot be attributed solely to tumor debulking surgery, as confounding by indication is the most likely explanation. In supraglottic laryngeal SCC, one study reported a local recurrence-free survival rate and overall survival of 80% and 88%, respectively, in 25 cases compared with 86% and 77%, respectively, in 24 controls. In sarcomas, local recurrence-free survival was 25%, 65% and 100% for unknown resection margins, wide local excision and radical excision, respectively. **Conclusions**: The heterogeneous data indicate the need for higher-quality research. It would be interesting to investigate whether an attempt to surgically debulk paranasal sinus tumors while preserving adjacent vital organs (e.g., the orbit and/or brain) should be incorporated into the treatment strategy for patients with extended paranasal sinus squamous cell carcinoma. Tumor debulking surgery did not improve survival in supraglottic laryngeal SCC or head and neck soft tissue sarcomas.

## Simple Summary

In this systematic review, we examined whether tumor debulking surgery followed by non-surgical treatment improves survival compared with non-surgical treatment alone in patients with head and neck cancer. Three small retrospective case–control studies suggested a potential survival benefit following tumor debulking for extended paranasal squamous cell carcinoma. However, these findings should be interpreted with caution because of the heterogeneity in the available data. Further, the observed survival benefits cannot be attributed solely to tumor debulking surgery, most likely due to confounding by indication. Nevertheless, it would be interesting to investigate whether an attempt to surgically debulk paranasal sinus tumors while preserving adjacent vital organs (e.g., the orbit and/or brain) should be incorporated into the treatment strategy for patients with extended paranasal sinus squamous cell carcinoma. Future research may also benefit from a clear definition and radiomics-based quantification of tumor debulking surgery.

## 1. Introduction

Surgery in patients with head and neck cancer (HNC) mainly addresses the primary tumor or the metastatic cervical lymph node disease. Following diagnostic surgery, tumor resection aims to excise the primary tumor with adequate safety margins. Researchers have, thus far, agreed on the complexity of defining the exact adequate safety margin [1,2,3,4,5]. Resection (R) status may be described as R_0_ (clear margins), R_1_ (microscopically positive margins) or R_2_ (macroscopically positive margins). The latter usually occurs with the intention of achieving an R_0_ resection, resulting in residual macroscopic disease (R_2_ resection) due to intraoperative findings.

When surgeons do not aim to achieve an R_0_ resection, the tumor may be unresectable. Indeed, a substantial proportion of head and neck tumors already present as unresectable at the time of initial diagnosis. Such tumors might invade the prevertebral space, internal carotid artery, masticator space, pterygoid muscles, mediastinal structures and other areas [6]. In such cases, primary tumor resection may be considered possible only at the cost of sacrificing critical anatomical structures.

The exact proportion of unresectable tumors depends largely on the tumor’s site or the involvement of critical anatomical structures. Orbital involvement has been reported in 30% to 50% of paranasal sinus malignancies [7]. Approximately 20% of patients with paranasal sinus malignancies present with brain invasion [8]. About 3% to 7% of HNCs invade the prevertebral space [9]. Encasement or direct invasion of the common or internal carotid artery is observed in 2% to 7% of HNCs [10].

Another option is tumor debulking surgery, the first cousin of R_2_ resection [11,12]. The latter is a procedure whereby a surgically incurable malignant neoplasm is partially removed without curative intent to improve the effectiveness of subsequent systemic treatment, radiotherapy or other adjunctive modalities, thereby improving survival [13]. In the head and neck, debulking surgery usually aims to alleviate patients’ symptoms; the most common case is laryngeal (or even hypopharyngeal) debulking surgery to relieve airway obstruction and dyspnea [14].

For unresectable primary or neck metastatic head and neck tumors, non-surgical treatment (i.e., radiotherapy, chemotherapy, immunotherapy or their combinations) is usually the treatment of choice. Debulking surgery before non-surgical treatment is generally not considered to improve survival in HNC. On the contrary, debulking surgery is widely used in ovarian cancer [15]. Theoretical arguments in favor of debulking surgery include reduced radiation fields [13], improved drug delivery by removing large necrotic masses [15] and increased chance of removing treatment-resistant tumor clones [16]. Furthermore, debulking of laryngeal or even hypopharyngeal tumors helps avoid tracheostomy [17], which increases the risk for stoma recurrence [18] and results in reduced survival.

In this systematic review, our primary aim was to map the available evidence on the effect of non-palliative tumor debulking surgery on survival in patients with HNC. Our secondary aim was to assess the potential survival benefit of this approach by examining whether tumor debulking surgery followed by non-surgical treatment improves survival compared with non-surgical treatment alone in patients with HNC.

## 2. Materials and Methods

We conducted this systematic review in accordance with the Preferred Reporting Items for Systematic Reviews and Meta-Analyses (PRISMA) guidelines [19]. Additionally, the review protocol was prospectively registered (registration number: CRD42024613312) with the International Prospective Register of Systematic Reviews (PROSPERO).

### 2.1. Search Strategy

From 1 December 2024 to 10 December 2024, A.I.G. and M.S. independently searched the databases PubMed, Scopus and the Cochrane Central Register of Controlled Trials (CENTRAL) for published studies using the following keywords: “debulking”, “oral”, “oropharyngeal”, “nasopharyngeal”, “nasal”, “paranasal”, “sinus”, “laryngeal”, “hypopharyngeal”, “tonsillar”, “tongue”, “tongue base”, “neck”, “lymph node”, “cancer”, “carcinoma” and “disease”. Initially, A.I.G. and M.S. independently screened the titles and abstracts of all retrieved articles for eligibility (see the Supplemental File S1 for the full search strategy).

### 2.2. Eligibility Criteria

The inclusion criteria were human studies or case series in English or German that reported survival outcomes in patients undergoing debulking surgery for malignant head and neck tumors followed by non-surgical treatment.

The exclusion criteria were animal or cadaveric studies, case reports, studies in languages other than English or German, studies referring to non-surgical tumor debulking using non-surgical treatments (e.g., tumor debulking by radiotherapy, chemotherapy, immunotherapy, photodynamic therapy), studies describing surgical tumor debulking without mentioning survival, studies assessing debulking surgery not followed by non-surgical treatment, studies evaluating debulking surgery as a palliative procedure and studies investigating thyroid cancer, skin cancer, metastatic disease to the head and neck of primary tumors other than primary HNC, lymphomas, benign tumors and tumors characterized neither as benign nor as malignant (e.g., paragangliomas/pheochromocytomas) [20].

### 2.3. Study Selection

After screening the titles and abstracts, A.I.G. and M.S. independently reviewed the full-text articles and selected studies for inclusion based on the eligibility criteria. Additionally, A.I.G. and M.S. independently screened the reference list of all included studies to identify additional eligible studies. Any discrepancies between the two reviewers were resolved by E.T., D.D. and B.H. through the majority criterion.

### 2.4. Assessment of Study Quality

We assessed the level of evidence according to the Oxford 2011 Levels of Evidence Table [21]. We evaluated the quality of non-randomized studies (case–control or cohort studies) with the Newcastle–Ottawa Scale. The Newcastle–Ottawa Scale consists of three domains with a total score of 9. A total score above 7 is considered an indicator of a high-quality study [22,23].

### 2.5. Data Extraction and Analysis

A.I.G. and M.S. independently extracted and recorded the following data from the included studies: year of publication, time period, country, study design, HNC histologic type, HNC site, HNC disease phase (i.e., primary or recurrency), tumor (T) and nodal (N) classification, number of subjects undergoing tumor debulking surgery of the primary tumor or the nodal disease, definition of tumor debulking surgery, volume of residual tumor, number of subjects undergoing non-surgical treatment (e.g., radiation, chemotherapy, immunotherapy) after debulking surgery, type of non-surgical treatment without previous debulking surgery, number of subjects that underwent non-surgical treatment without previous debulking surgery, local recurrence-free survival or local recurrence-free survival rate (LR), disease-free survival (DFS) and overall survival (OS).

In the case of missing data, studies were included only if they reported all the following: HNC histologic type, site and phase, number of subjects undergoing debulking surgery of the primary tumor or nodal disease, and at least one survival outcome, i.e., LR-free survival, DFS or OS.

Subjects receiving non-surgical treatment (e.g., radiotherapy, chemotherapy, immunotherapy) after debulking surgery were considered cases. Subjects receiving non-surgical treatment without previous debulking surgery were considered controls. Tumor debulking surgery was defined as surgery without removal of all macroscopic disease.

## 3. Results

### 3.1. Literature Search

**Figure 1:**
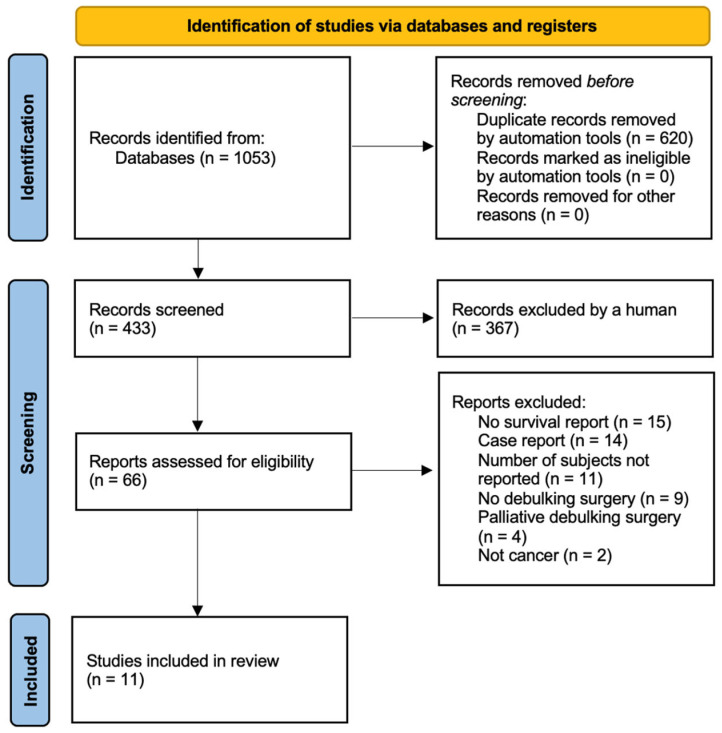
Prisma flow diagram.

The literature search using all keyword combinations yielded 1053 articles, of which 433 remained after deduplication. Title and abstract screening resulted in 66 potentially relevant articles. Finally, 11 articles met the eligibility criteria (Figure 1).

### 3.2. Study Characteristics

The studies were published between 1998 and 2015 (Table 1). Data were retrospectively collected from 1970 to 2014. More than half of the studies were conducted in the United States of America [24,25,26] and Japan [27,28,29]. Only four studies were case–control studies [27,29,30,31], whereas the majority were case series [24,25,26,28,32,33]. Only one study investigated residual tumor volume (Table 1) [28].

### 3.3. Tumor Subsite, TNM Stage and Histology

Most studies (6/11) investigated tumors of one or more paranasal sinuses [26,27,28,30,31,34]. Several other head and neck subsites (i.e., oropharynx, oral cavity, nasopharynx and hypopharynx) were not represented. Only two studies focused on outcomes of recurrent neck metastatic disease [25,32], whereas the majority (7/11) evaluated primary HNC (Table 2) [24,26,27,28,29,30,31]. Only three studies explicitly reported T-stage [26,27,28], information that was not easily extracted. Five studies focused specifically on squamous cell carcinoma (SCC) [25,27,28,29,33], whereas five studies reported a mixed population of between two and 10 different histologic types [26,30,31,32,34]. Esposito and coauthors reported four subjects with different tumor types, including one benign tumor (i.e., giant cell tumor, which was included due to its locally destructive behavior) [26]. Including their study was a post hoc, justified deviation from the registered PROSPERO protocol. Barker and coauthors reported outcomes of adult tissue sarcomas only (Table 2) [24]. No study mentioned HPV status.

### 3.4. Definition of Tumor Debulking Surgery

Most studies (7/11) defined tumor debulking surgery as either “subtotal removal (or resection)” [24,26,31] or “maximal surgical debulking” [29,33,34]. Two studies characterized tumor debulking surgery as “macroscopic surgical clearance” or “removal of all macroscopic tumor” (Table 3) [30,32].

### 3.5. Survival in Cases and Controls

Data from three case–control studies suggested longer survival in subjects with various paranasal sinus malignancies who underwent radiotherapy after debulking surgery (cases) than in subjects with various paranasal sinus malignancies who received radiotherapy alone (controls; Table 4) [27,30,31].

Itami and coauthors reported a higher LR-free survival rate (46%) in 17 cases with SCC of the maxillary sinus than in five controls with SCC of the maxillary sinus (0%) [27]. Furthermore, Jansen and coauthors reported longer survival in 50 cases with various paranasal sinus malignancies than in 18 controls with various paranasal sinus malignancies. In that study, LR, DFS and OS rates were 65%, 53% and 60% in cases versus 47%, 6% and 9% in controls, respectively [30]. Lastly, Vedrine and coauthors reported a median OS of 108 months in five cases with various sphenoid malignancies compared with 13 months in 17 controls with various sphenoid malignancies [31].

In the only study that exclusively examined cases of subjects with T1 or T2 supraglottic laryngeal SCC, Suzuki and coauthors reported a lower LR-free survival rate in 25 cases (80%) than in 24 controls (86%). Moreover, the authors reported higher OS in cases (88%) than in controls (77%) [29].

Data from case series were less revealing than those from case–control studies. Stafford and coauthors (eight subjects) [32] and Nutting and coauthors (72 subjects) [33] investigated survival outcomes in subjects with predominantly SCC recurrent neck metastatic disease who underwent brachytherapy after tumor debulking surgery. Stafford and coauthors reported LR, DFS and OS of 14.3, 13.9 and 20.5 months, respectively [32], whereas Nutting and coauthors reported 5-year DFS and OS rates of 11% and 23%, respectively [33]. Furthermore, Kawashima and coauthors reported a two-year LR-free survival rate of 62% in 43 subjects with SCC of the maxillary sinus who underwent radiotherapy after debulking surgery. Notably, the authors reported that gross residual tumor volume was a significant independent prognostic factor for LR (*p* = 0.002). Specifically, the LR-free survival rate was higher in 36 subjects with a gross residual tumor volume less than 40 cm^3^ (69% at two years) than in eight subjects with a gross residual tumor volume larger than 40 cm^3^ (31% at one year; *p* < 0.001) [28].

### 3.6. Quality Assessment

We assessed the quality of the four case–control studies only [27,29,30,31]. The study by Jansen and coauthors was of high quality (7/9) [30] on the Newcastle–Ottawa Scale, whereas the remaining three studies were of moderate quality, reaching 5/9 [27], 6/9 [29] and 6/9 [31] (Table 5). The main limitation of these four studies was the lack of homogeneity among subjects assigned to the case group, i.e., subjects with HNC undergoing debulking surgery followed by non-surgical treatment.

## 4. Discussion

Unresectable primary and unresectable regionally metastatic HNC are usually addressed with radiotherapy and systemic treatment. Despite recent relaxation of their definition [35,36,37], surgical treatment of unresectable tumors is commonly avoided because of either the difficulty of achieving clear surgical margins or the resulting morbidity. Otorhinolaryngologists may encounter tumors in which safety margins can only be assessed intraoperatively. This might lead to hesitation in indicating surgery, which, in turn, may result in a lower rate of complete resections. The presumed value of debulking surgery could potentially increase the number of complete resections. However, in contrast to other cancer types (e.g., ovarian cancer) [15], debulking surgery before non-surgical treatment is not considered to improve survival in HNC. Debulking surgery may be defined as “reducing the tumor volume by surgery and leaving tumor tissue behind.” In this systematic review, our primary aim was to map the available literature data, while our secondary aim was to investigate whether tumor debulking surgery followed by non-surgical treatment improves survival compared with non-surgical treatment alone in patients with HNC.

For this reason, we used multiple keywords in the literature search. The keywords included all head and neck subsites, as well as more specific anatomic subsites such as the tonsil, tongue base and tongue. A crucial keyword was “debulking”, which led to several challenges. First, we identified and excluded multiple studies in which debulking was defined as “tumor shrinkage after radiotherapy or systemic treatment.” In addition, “debulking” was used as either “piecemeal, but still complete sinonasal tumor resection” or “palliative procedures performed to relieve symptoms” in nine and four studies, respectively. These 13 studies were excluded during the eligibility assessment. These findings suggested a lack of consistent and specific use of the word “debulking” in the literature.

To further refine the search, we used “macroscopic” as a complementary keyword to “debulking”, as several articles used the phrase “with or without macroscopic disease”. This led to the screening of articles describing the survival benefit of including surgical resection of cT4_b_ SCC paranasal sinus tumors [38,39] and cT4_b_ non-SCC paranasal sinus tumors [40] in the treatment plan compared with non-surgical treatment alone. However, these studies did not refer to debulking surgery, but rather to complete surgical resection in selected cT4_b_ cases. Thus, they were not included in the review.

The included studies were either case–control or case-series. The four case–control studies were of moderate quality according to the Newcastle–Ottawa Scale. The main limitations were related to the definition of cases and controls. Most commonly, the definition of subjects undergoing debulking surgery was unclear within studies and heterogeneous between studies (e.g., subtotal resection, with macroscopic disease, positive margins, maximal tumor resection with preservation of the bony framework). This high degree of heterogeneity limited the generalizability of our results and reduced the strength of our findings [41,42].

The latter highlighted the need for a precise definition of tumor debulking surgery. One possible definition could be “the maximal possible partial resection of inoperable tumors without jeopardizing function before non-surgical treatment”. This definition should be tailored to the affected head and neck subsite. For example, debulking surgery of a cT_4b_ paranasal sinus or nasopharyngeal tumor should not compromise the function of adjacent vital organs, such as the brain. However, this definition would not apply to smaller tumors. In the case of a T_3_ supraglottic laryngeal tumor planned for primary radiochemotherapy—where the tumor could otherwise be adequately managed with total laryngectomy—would tumor debulking surgery be meaningful for primary radiochemotherapy to be more effective? This consideration would modify the previous definition as follows: “The maximal possible partial resection of tumors without jeopardizing function before non-surgical treatment”.

One disadvantage of this definition is the lack of quantification. The only study that quantified the postoperative residual tumor volume on computed tomography (CT) scans during the planning of radiotherapy after tumor debulking surgery in subjects with paranasal sinus tumors was that by Kawashima and coauthors [28]. They suggested a survival benefit in subjects with less than 40 cm^3^ residual tumors who received radiotherapy doses of more than 60 Gy. Despite notable methodological limitations, the authors managed to quantify the extent of tumor debulking surgery. Recent advances in radiomics have enabled more precise calculation of HNC tumor volume and its prognostic value [43,44] and may help standardize the extent or degree of tumor debulking surgery in future studies.

This heterogeneity further extended to the tumor subsite. No study investigated tumors of the oral cavity, oropharynx, nasopharynx and hypopharynx. Furthermore, subjects undergoing debulking surgery were not analyzed separately but were included as part of mixed populations. Moreover, the survival data may not have corresponded directly to the subjects undergoing debulking surgery. TNM stage was rarely reported, with only three studies providing information for the T-stage. Finally, approximately half of the studies included populations with mixed histologic types, ranging from two to 10 different histologic types, without survival stratification by histology. HPV status, a significant factor in treatment planning and survival [45], was not reported. These limitations applied to the control subjects and the case series studies. The lack of standardized reporting and objective metrics, such as residual tumor volume after debulking surgery, precluded us from performing a meta-analysis. These findings imply that conclusions should not be generalized to specific subgroups without further evidence.

Our search identified three retrospective, small-sample, case–control studies of moderate quality. These reported longer survival in patients with paranasal sinus SCC who underwent radiotherapy following debulking surgery than in subjects with paranasal sinus SCC who underwent radiotherapy alone [27,30,31]. In addition, a case-series study of subjects with paranasal sinus SCC undergoing radiotherapy reported that lower residual tumor volume after tumor debulking surgery was associated with longer local recurrence-free survival [28]. However, these observed survival benefits cannot be attributed solely to tumor debulking surgery, given the multimodal treatments and the lack of adjustment for potential confounding factors. Confounding by indication is the most likely explanation. Patients who received non-surgical treatment only, i.e., control patients, probably differed systematically in operability and general condition. Therefore, these conclusions should be critically and carefully assessed.

Nevertheless, it would be interesting to investigate whether an attempt to surgically debulk paranasal sinus tumors while preserving adjacent vital organs (e.g., the orbit and/or brain) should be incorporated into the treatment strategy for patients with extended paranasal sinus SCC.

The other relevant studies were two case series of patients with unresectable metastatic neck disease. Stafford [32] and Nutting [33] suggested that neck debulking surgery followed by brachytherapy may be a reasonable treatment approach. However, the absence of control groups in these studies significantly limited the interpretability and clinical value of their findings.

Additional data worth mentioning originated from the case–control study by Suzuki and coauthors. The authors reported similar, if not lower, LR-free survival in 25 cases with T1 or T2 supraglottic LSCC and in 24 controls. Nevertheless, OS was higher in the cases (88%) than in the controls (77%). These findings suggested a limited role of debulking surgery in improving survival in laryngeal supraglottic SCC. Furthermore, in their case series, Barker and coauthors reported that the LR-free survival rate was strongly correlated with the extent of surgical excision in subjects with primary head and neck soft tissue sarcoma, with rates of 25% for debulking surgery, 65% for wide local excision and 100% for radical excision [24]. These data also suggested a limited role of debulking surgery in the survival of head and neck soft tissue sarcomas. Overall, these data indicated a strong dependence of debulking surgery on tumor site and histology.

One main concern when investigating the role of debulking surgery is the well-established association between R1 resection and reduced DFS and OS [46,47]. This raises the following question. Given that R_1_ resection results in residual microscopic disease and worse outcomes, why should the impact of even greater residual disease—i.e., debulking surgery with macroscopic residual tumor—be investigated at all? First, piecemeal resection of paranasal sinus malignancies, which nowadays constitutes common practice due to endoscopic sinus surgery [48], does not allow reliable assessment of resection status, i.e., R-status, in paranasal sinus malignancies. Second, existing data on the adverse prognostic impact of R_1_ resection derive primarily from intended R_0_ resection. On the contrary, the effect of an intended R_2_ resection, or tumor debulking surgery, has not been adequately studied so far in large unresectable tumors, traditionally managed with primary radiotherapy or chemoradiotherapy. The potential for organ preservation or improved survival justifies further investigation into the role of intended R_2_ resection or tumor debulking surgery in the survival of patients with HNC.

Randomized controlled trials and cohort studies were not identified in this review. Moreover, our search did not identify any systematic reviews investigating the role of debulking surgery in specific histologic types. Based on the level of evidence (review of case–control studies), this systematic review may be considered level 4, as the included case–control studies were heterogeneous. Among the included studies, radiotherapy was the most commonly used non-surgical treatment. Other treatment modalities, including neoadjuvant or adjuvant chemotherapy and immunotherapy, were either not reported or used too infrequently to permit meaningful analysis. Nevertheless, the use of the broad term “non-surgical treatment” is highly nonspecific since it pools multiple treatments such as radiotherapy, brachytherapy, chemotherapy, immunotherapy and phototherapy. These treatments have distinct mechanisms of action and different outcomes. Grouping these modalities under a single umbrella of “non-surgical treatment” weakens the comparison between debulking surgery followed by non-surgical treatment and non-surgical treatment alone. A more specific comparison, e.g., debulking surgery followed by radiotherapy and radiotherapy alone, would likely provide more clinically meaningful evidence.

The main limitation of this review was the absence of randomized controlled trials, cohort studies and well-designed, homogeneous, prospective, large case–control studies. Furthermore, the heterogeneous case–control studies and case series in this review could not provide any pathophysiological explanation for the potential value of debulking surgery in HNC. Recently, Hishida and coauthors suggested a potential survival benefit for patients in whom a synergetic effect of debulking surgery and systemic/radiation therapy may be expected [12]. Debulking a large proportion of resting cells during the cell cycle might enhance the effectiveness of systemic therapy [13,49]. Reducing tumor volume might also be beneficial by decreasing the radiation field and enhancing the therapeutic effect of radiotherapy [12]. However, Hishida and coauthors referred to cancers other than HNC.

Further limitations extended to the general characteristics of the subjects. Age [50], comorbidities [51] and concomitant medication were neither reported nor analyzed. Zanoni and coauthors concluded that age and comorbidities were independent survival factors in subjects with oral cavity cancer [52]. The lack of such information was a major source of residual confounding. Furthermore, Verdonck-de Leeuw and coauthors reported that higher health-related quality of life was associated with longer OS [53]. This finding highlighted the importance of documenting quality of life in HNC databases. The studies included in this review failed to assess whether prolonged survival corresponded to meaningful, high-quality survival. Also, we did not include specific histologic types (e.g., adenoid cystic carcinoma, squamous cell carcinoma, adenocarcinoma) as separate keywords for pragmatic reasons.

A further significant limitation of the studies in the current review was the period during which they were published. Data were collected between 1970 and 2014 (Table 1). The documentation and medical practice of that period do not reflect current advantages in treatment planning and equipment. The most recent study included in this review was published 10 years ago. Since then, surgery and radiotherapy have continued to advance, reducing morbidity, while the use of targeted treatments, such as immunotherapy, has grown rapidly [54,55]. This further limits the applicability of the findings of the present review.

An additional limitation concerned the purpose of the surgery performed in each study. All studies were retrospective. Therefore, it cannot be excluded that, rather than intentional true debulking surgery, the true aim of surgery was complete tumor resection, with intraoperative findings revealing that the tumor was unresectable, resulting in an R_2_ resection. This would differentiate intentional debulking surgery from an unintended R_2_ resection. It also indicates a blurred distinction between maximal safe resection and true debulking. The theoretical rationale for tumor debulking surgery (i.e., improved radiation targeting, drug delivery and removal of resistant clones) is largely extrapolated from ovarian cancer and remains unproven in HNC.

Considering the absence of well-designed studies and the presence of a higher risk of bias, this systematic review would be classified as level 4 evidence. The heterogeneous data on histology, tumor subsite, TNM stage and adjuvant treatment, and the lack of adjustment for confounding factors, did not allow for an accurate estimation of the true benefit of tumor debulking surgery. The lack of adjustment for confounding factors [56] and standardization [57] might have led to an overestimation of the effect size, while the relatively small sample size pooled across the case–control studies could have resulted in an underestimation of the effect size [58]. Methodological heterogeneity and variations in the definition of tumor debulking surgery might have also affected the accuracy of the synthesized results. Based on these observations, this review should be considered hypothesis-generating. The data presented here are insufficient to support any practice-changing or clinical decision-making implications.

Nevertheless, the main contribution of this systematic review is highlighting the need for higher-quality, contemporary research on this topic. Future prospective studies should focus on a specific histologic type, preferably SCC because of its higher prevalence. Studies should ideally be randomized to minimize the effect of potential confounding factors. Future studies should also use a standardized treatment protocol, such as radiochemotherapy following tumor debulking surgery.

Tumor debulking surgery should be standardized and quantified. Quantification could be performed using CT or magnetic resonance imaging scans during radiotherapy planning, either by measuring the absolute residual tumor volume or the relative (percentage) residual tumor volume. The same imaging modality should be used pre- and postoperatively to allow direct comparison of the effect of tumor debulking surgery on tumor volume. Challenges would include accurately delineating potentially irregular tumor margins. This might be aided by using positron emission tomography/CT scans [59]. Deep learning-based tumor volume delineation has the potential to assist clinical target volume delineation in radiation oncology [60]. Furthermore, deep learning-based automated segmentation would reduce inter-rater variability by improving consistency compared with manual contouring. Moreover, PET/CT tumor-volume-derived imaging biomarkers could be developed [60]. However, accurate delineation would remain challenging because of postoperative changes, edema and the complex anatomy of the head and neck region.

It would be of particular interest to determine whether a specific residual tumor threshold should exist for a procedure to be characterized as “tumor debulking surgery”. Such criteria should be clearly defined. Lastly, it remains unclear which tumor subsite should be investigated first. Based on the hypothesis generated by this review, an appropriate starting point could be patients with unresectable paranasal sinus tumors, using clearly defined criteria. Surgeons should aim to debulk the tumor by avoiding injury to adjacent critical organs (i.e., skull base). Another potential subsite could be the larynx or hypopharynx. In these patients, it would be interesting to investigate whether tumor debulking surgery before primary radiochemotherapy improves survival compared with primary radiochemotherapy alone. Studies should be adequately powered and multicenter. Outcomes should include local recurrence-free survival, disease-free survival, overall survival and quality of life measures.

## 5. Conclusions

This systematic review highlighted the need for higher-quality research on the value of tumor debulking surgery. The potential survival benefits observed after tumor debulking surgery for paranasal sinus malignancies were challenged by the heterogeneous, low-quality data derived from retrospective, small-sample studies and cannot be attributed solely to tumor debulking surgery, as confounding by indication is the most likely explanation. Furthermore, the included studies were published between 1998 and 2015, with data collected from 1970 to 2014, and the most recent study was published a decade ago. Therefore, these data predate contemporary radiotherapy techniques and the routine use of immunotherapy, which further limits the applicability of these findings to current practice. Nevertheless, it would be interesting to investigate whether an attempt to surgically debulk paranasal sinus tumors while preserving adjacent vital organs (e.g., the orbit and/or brain) should be incorporated into the treatment strategy for patients with extended paranasal sinus SCC.

## Figures and Tables

**Table 1:** Study characteristics.

Study	Date	Data Collection	Country	Study Type ^2^	Evidence Level
Stafford [32]	1998	n/a ^1^	United Kingdom	case series	IV
Itami [27]	1998	1973–1992	Japan	case–control	III
Jansen [30]	2000	1977–1996	The Netherlands	case–control	III
Kawashima [28]	2001	1992–1999	Japan	case series	IV
Barker [24]	2003	1970–2000	United States of America	case series	IV
Esposito [26]	2006	Until 2003	United States of America	case series	IV
Nutting [33]	2006	1997–2003	United Kingdom	case series	IV
Vedrine [31]	2009	1998–2004	France	case–control	III
Choe [25]	2011	1986–2006	United States of America	case series	IV
Suzuki [29]	2014	1990–2011	Japan	case–control	III
Caesar [34]	2015	1998–2014	The Netherlands	case series	IV

^1^ Not available; ^2^ all studies were retrospective.

**Table 2:** Case characteristics.

Study	Subsite	Histology	Phase	T-Status			
				T1	T2	T3	T4
Stafford [32]	neck	SCC ^1^	recurrency	n/p	n/p	n/p	n/p
		Adenocarcinoma					
Itami [27]	maxillary sinus	SCC ^1^	primary	0	1	3	13
Jansen [30]	paranasal sinuses	SCC ^1^	primary	n/a	n/a	n/a	n/a
		Adenocarcinoma					
		ACC ^2^					
		SNUC ^3^					
Kawashima [28]	maxillary sinus	SCC ^1^	primary	0	5	12	26
Barker [24]	head and neck	Sarcoma	primary	n/a	n/a	n/a	n/a
Esposito [26]	sphenoid	Neuroendocrine carcinoma	primary	0	0	0	4
		SNUC ^3^					
		Mucoepidermoid carcinoma					
		Giant cell tumor ^4^					
Nutting [33]	neck	Mainly SCC ^1^	recurrency	n/p	n/p	n/p	n/p
Vedrine [31]	sphenoid	Lymphoma	primary	n/a	n/a	n/a	n/a
		ACC ^2^					
		SCC ^1^					
		Adenocarcinoma					
		SNUC ^3^ and others					
Choe [25]	head and neck	SCC ^1^	recurrency	n/a	n/a	n/a	n/a
Suzuki [29]	larynx	SCC ^1^	primary	n/a	n/a	n/a	n/a
Caesar [34]	paranasal sinuses	SCC ^1^	recurrency	n/a	n/a	n/a	n/a
		Adenocarcinoma					
		SNUC ^3^					
		ACC ^2^					
		Sarcoma					

^1^ Squamous cell carcinoma; ^2^ adenoid cystic carcinoma; ^3^ sinonasal undifferentiated carcinoma; ^4^ benign tumor, included due to its locally destructive behavior; n/p: not applicable; n/a: not available.

**Table 3:** Definition of tumor debulking surgery.

Study	Subsite	Definition
Stafford [32]	neck	macroscopic surgical clearance or substantial debulking
Itami [27]	maxillary sinus	piecemeal tumor debulking; patients were considered to have macroscopic residual disease if the surgeon suspected a tumor remaining in the sinus
Jansen [30]	paranasal sinuses	removal of all macroscopic tumors without additional removal of noninvolved bony structures
Kawashima [28]	maxillary sinus	maximal piecemeal tumor debulking with preservation of the bony framework of the maxillary sinus
Barker [24]	head and neck	subtotal resection
Esposito [26]	sphenoid	subtotal removal
Nutting [33]	neck	maximal surgical debulking
Vedrine [31]	sphenoid	subtotal resection or debulking (macroscopically incomplete R2)
Choe [25]	head and neck	tumor debulking when possible as a form of cytoreduction
Suzuki [29]	larynx	to reduce tumor volumes to the smallest extent possible
Caesar [34]	paranasal sinuses	maximal debulking

**Table 4:** Survival data.

Study	Subsite	Cases	NST ^1^				Controls			
		n		LR ^5,6^	DFS ^5,7^	OS ^5,8^	n	LR ^5,6^	DFS ^5,7^	OS ^5,8^
Stafford [32]	neck	8	BT ^9^	14.3	13.9	20.5	n/a	n/a	n/a	n/a
Itami [27]	MS ^2^	17	RT ^10^	46%	-	-	5	0%	-	-
Jansen [30]	PNS ^3^	50	RT ^10^	65%	53%	60%	18	47%	6%	9%
Kawashima [28] ^12^	MS ^2^	43	RT ^10^	62%	-	-	n/a	n/a	n/a	n/a
Barker [24]	H&N ^4^	4	RT ^10^	25%	-	-	n/a	n/a	n/a	n/a
Esposito [26]	sphenoid	4	RT ^10^	-	-	21	n/a	n/a	n/a	n/a
Nutting [33]	neck	72	BT ^9^	-	11%	23%	n/a	n/a	n/a	n/a
Vedrine [31]	sphenoid	5	RT ^10^	-	-	108	17	-	-	13
Choe [25]	H&N ^4^	19	RCTH ^11^	-	-	^	n/a	n/a	n/a	n/a
Suzuki [29]	larynx	25	RT ^10^	80%	-	88%	24	86%	-	77%
Caesar [34]	PNS ^3^	12	PT ^13^	-	-	36 + 5 ^14^	n/a	n/a	n/a	n/a

^1^ Type of non-surgical treatment following debulking surgery; ^2^ maxillary sinus; ^3^ paranasal sinuses; ^4^ head and neck; ^5^ after debulking surgery, either in months or in percentage after five years of follow-up, unless otherwise mentioned; ^6^ local recurrence in months or local recurrence-free survival after five years in percentage, unless otherwise mentioned; ^7^ disease-free survival; ^8^ overall survival; ^9^ brachytherapy; ^10^ radiotherapy; ^11^ chemoradiotherapy; ^12^ after two years of follow-up; ^13^ photodynamic therapy; ^14^ 36 months with long follow-up and 5 months alive without long follow-up; ^: increased compared to non-surgical treatment; n/a: not available.

**Table 5:** Quality assessment of case–control studies by the Newcastle–Ottawa Scale.

	Itami 1998 [27]	Suzuki 2014 [29]	Jansen 2000 [30]	Vedrine 2009 [31]
Selection				
Is the case definition adequate? (maximum 1 *)				
Representativeness of the cases (maximum 1 *)				
Selection of controls (maximum 1 *)	*	*	*	*
Definition of controls (maximum 1 *)	*	*	*	*
Comparability				
Comparability of cases and controls based on the design or analysis (maximum 2 *)		*	**	*
Exposure				
Ascertainment of exposure (maximum 1 *)	*	*	*	*
Same method of ascertainment for cases and controls (maximum 1 *)	*	*	*	*
Non-response rate (maximum 1 *)	*	*	*	*
Total (out of 9 *)	5	6	7	6
Quality	Moderate	Moderate	High	Moderate

* The presence of * under a study (i.e., Itami 1998 [27], Suzuki 2014 [29], Jansen 2000 [30], Vedrine 2009 [31]) indicated that the condition of the first column has been met in this study.

## Data Availability

The data of this study are available upon reasonable request from the corresponding author.

## Supplementary Materials

The following supporting information can be downloaded at: https://www.mdpi.com/article/10.3390/curroncol33070409/s1, Supplemental File S1: Full search Strategy Round2.

## Abbreviations

The following abbreviations are used in this manuscript:

HNC	Head and neck cancer
R	Resection
R_0_	Resection with clear margins
R_1_	Resection with unclear microscopic margins
R_2_	Resection with unclear macroscopic margins
PRISMA	Preferred Reporting Items for Systematic Reviews and Meta-Analyses
T	Tumor
N	Nodal
LR	Local recurrence
DFS	Disease-free survival
OS	Overall survival
SCC	Squamous cell carcinoma
