# Supplementary material for: The Role of Tumor Debulking Surgery in Improving Survival of Patients with Head and Neck Cancer: A Systematic Review
Source: Curr Oncol. 2026 Jul 9;33(7):409. doi: 10.3390/curroncol33070409 (PMC13409472; doi:10.3390/curroncol33070409)
Supplement: Supplementary file 1 [file curroncol-33-00409-s001.zip › Supplemental File S1.pdf]

**Database**	**Complete search string**	**Filters and limits**
PubMed	(<br>debulk*[tiab]<br>OR cytoreduct*[tiab]<br>OR "tumor reduction"[tiab]<br>OR "tumour reduction"[tiab]<br>OR "cytoreductive surgery"[tiab]<br>OR "tumor debulking"[tiab]<br>)<br>AND<br>(<br>"Head and Neck Neoplasms"[Mesh]<br>OR "head and neck cancer"[tiab]<br>OR "head and neck carcinoma"[tiab]<br>OR oropharyn*[tiab]<br>OR hypopharyn*[tiab]<br>OR laryn*[tiab]<br>OR nasopharyn*[tiab]<br>OR oral[tiab]<br>OR tonsil*[tiab]<br>OR tongue[tiab]<br>OR "base of tongue"[tiab]<br>)<br>AND<br>(<br>cancer[tiab]<br>OR carcinoma[tiab]<br>OR neoplasm*[tiab]<br>OR malignan*[tiab]<br>)<br>AND<br>(english[lang] OR german[lang])	No date restriction. Filters: English and German language
Scopus	TITLE-ABS-KEY(<br>debulk*<br>OR cytoreduct*<br>OR "tumor reduction"<br>OR "tumour reduction"<br>)<br>AND<br>TITLE-ABS-KEY(<br>("head" W/1 "neck")<br>OR oral<br>OR oropharyn*<br>OR hypopharyn*<br>OR laryn*<br>OR nasopharyn*<br>OR tonsil*<br>OR tongue<br>OR "base of tongue"<br>)<br>AND<br>TITLE-ABS-KEY(<br>cancer<br>OR carcinoma<br>OR neoplasm*<br>OR malignan*<br>)<br>AND (LANGUAGE(English) OR LANGUAGE(German))	No date restriction. Filters: English and German language
CENTRAL	(<br>debulk* OR cytoreduct* OR "tumor reduction" OR "tumour reduction" OR "cytoreductive surgery"<br>):ti,ab,kw<br>AND<br>(<br>"head and neck cancer" OR "head and neck carcinoma"<br>OR oropharyn* OR hypopharyn* OR laryn* OR nasopharyn*<br>OR oral OR tonsil* OR tongue OR "base of tongue"<br>):ti,ab,kw<br>AND<br>(<br>cancer OR carcinoma OR neoplasm* OR malignan*<br>):ti,ab,kw	No date restriction. Filters: English and German language
